# 2021 trends in the treatment of patients with strabismus in Japan

**DOI:** 10.1007/s10384-024-01144-5

**Published:** 2024-12-16

**Authors:** Keiko Kunimi, Toshiaki Goseki, Sachiko Nishina, Takashi Negishi, Miho Sato

**Affiliations:** 1 Department of Ophthalmology, International University of Health and Welfare, Atami Hospital, 13-1 Higashi-kaigan-cho, Atami-City, Shizuoka 413-0012 Japan; 2 Department of Ophthalmology, Kitasato University, 1-15-1 Kitasato, Minami-ku, Sagamihara, Kanagawa 252-0374 Japan; 3 Division of Ophthalmology, National Center for Child Health and Development, 2-10-1 Okura, Setagaya-ku, Tokyo, 157-8535 Japan; 4 Department of Ophthalmology, Juntendo University School of Medicine, 2-1-1 Hongo, Bunkyo-ku, Tokyo, 113-8421 Japan; 5 Department of Ophthalmology, Hamamatsu University School of Medicine, 1-20-1 Handayama, Higashi-ku, Hamamatsu-City, Shizuoka 431-3192 Japan

**Keywords:** Botulinum toxin therapy, Epidemiology, National survey, Sagging eye syndrome, Strabismus

## Abstract

**Purpose:**

To clarify the actual status of strabismus surgery and botulinum toxin (BTX) therapy in Japan in the year 2021.

**Study design:**

Cross-sectional study.

**Methods:**

We conducted a national survey of strabismus treatment in 2021 using a questionnaire consistent with a previous 2013 survey, incorporating additional questions about BTX therapy.

**Results:**

Among the 378 responding institutions, strabismus surgery or BTX therapy, or both, was performed at 185 institutions (49%; total cases, 10,767). In 151 (40%), 32 (8%), and 2 (1%) institutions, surgery only, surgery and BTX therapy, and BTX therapy only were performed, respectively. The distribution of institutions where strabismus surgery was performed consisted of 4 prefectures, accounting for 48% of the total, whereas no strabismus surgery was performed at any institution in 3 prefectures. Although the highest percentage of patients (23%) was aged between 10 and 19 years, 48.2% of the patients were aged 20 years or older, and 17% of them were aged 60 years or older. Exotropia (XT) was the most common type of strabismus (55%) followed by esotropia (ET) (24%). In terms of complex surgeries, 80.2% (more than 100 cases) were performed at institutions with more than 100 cases. Of the 34 institutions where BTX therapy was performed, 52% were performed at a single institution, and 18% were performed at 2 institutions where no strabismus surgery was performed. Patients with scarring in the extraocular muscles, such as thyroid eye disease, were the most treated, followed by those with ET, who were mainly given injection treatment.

**Conclusion:**

Institutions where strabismus surgery and BTX therapy could be performed were concentrated and limited.

**Supplementary Information:**

The online version contains supplementary material available at 10.1007/s10384-024-01144-5.

## Introduction

A decrease in quality of life (QOL) due to strabismus and improvement in QOL with strabismus surgery have been reported [1]. Additionally, a high score on the postoperative psychosocial and functional subscales of the Adult Strabismus-20 score [2, 3] has been reported in patients who underwent strabismus surgery [4]. These findings indicate that strabismus surgery improves patient QOL and that understanding the actual status of strabismus treatment is important. Previous Japanese studies have reported a prevalence of strabismus of 0.3% to 1.3% in children aged 6–12 years, of 2.1% in individuals of all ages, and of 3.6% in adults [5–7]. In Japan, a nationwide questionnaire survey of strabismus surgery was conducted in 2013 [8]. Among 710 institutions that participated in the survey, 320 (45%) institutions reported performing 10,671 surgeries in a year. Since 2013, there have been changes in lifestyle. Moreover, treatment strategies have improved in Japan. Demographic changes—an aging population combined with a low birth rate, environmental changes, and increased use of digital devices due to the implementation of online classes and teleworking—have occurred. Furthermore, botulinum toxin (BTX) therapy for the extraocular muscles has become covered by insurance after the revision of the medical payment system in 2020. Considering this background, conducting another survey to investigate the changes in strabismus treatment was deemed necessary. We performed a nationwide survey of strabismus surgery and BTX in 2021 to clarify the regional distribution of institutions where strabismus treatment was performed, age distribution of patients, types of strabismus, methods of treatment, and spread of BTX and its indications.

## Methods

### Ethical considerations

The study was approved by the institutional review board of the International University of Health and Welfare, Atami Hospital, Shizuoka, Japan (21-A-203). The study adhered to the principles of the Declaration of Helsinki. Informed consent was not required as the data were anonymized and deidentified.

### Study design

This study was designed as a cross-sectional survey conducted in 2021 and targeting institutions accredited by the Japanese Ophthalmological Society Specialty Program and members of the Japanese Association for Strabismus and Amblyopia.

### Participants

Questionnaires were sent to 966 institutions and 720 ophthalmologists. Patients who underwent strabismus surgery and BTX therapy between April 2021 and March 2022 were included. One respondent per institution was selected.

### Data collection

The questionnaire included items on the total number of surgeries, operated eyes (monocular or binocular), anesthesia methods, sex, age, type of strabismus, and surgical methods. Regarding BTX therapy, data on the number of injections, recipient eyes, sex and age, type of strabismus, and administration methods were collected. Detailed questionnaire descriptions are available in Online Resource 1–2.

### Data analysis

Institutions were categorized by the number of surgeries performed (1–9, 10–49, 50–99, and ≥ 100) and by prefecture; the status of strabismus treatment was summarized.

## Results

### Survey results from 2021

#### Distribution of cases and institutions

**Fig. 1:**
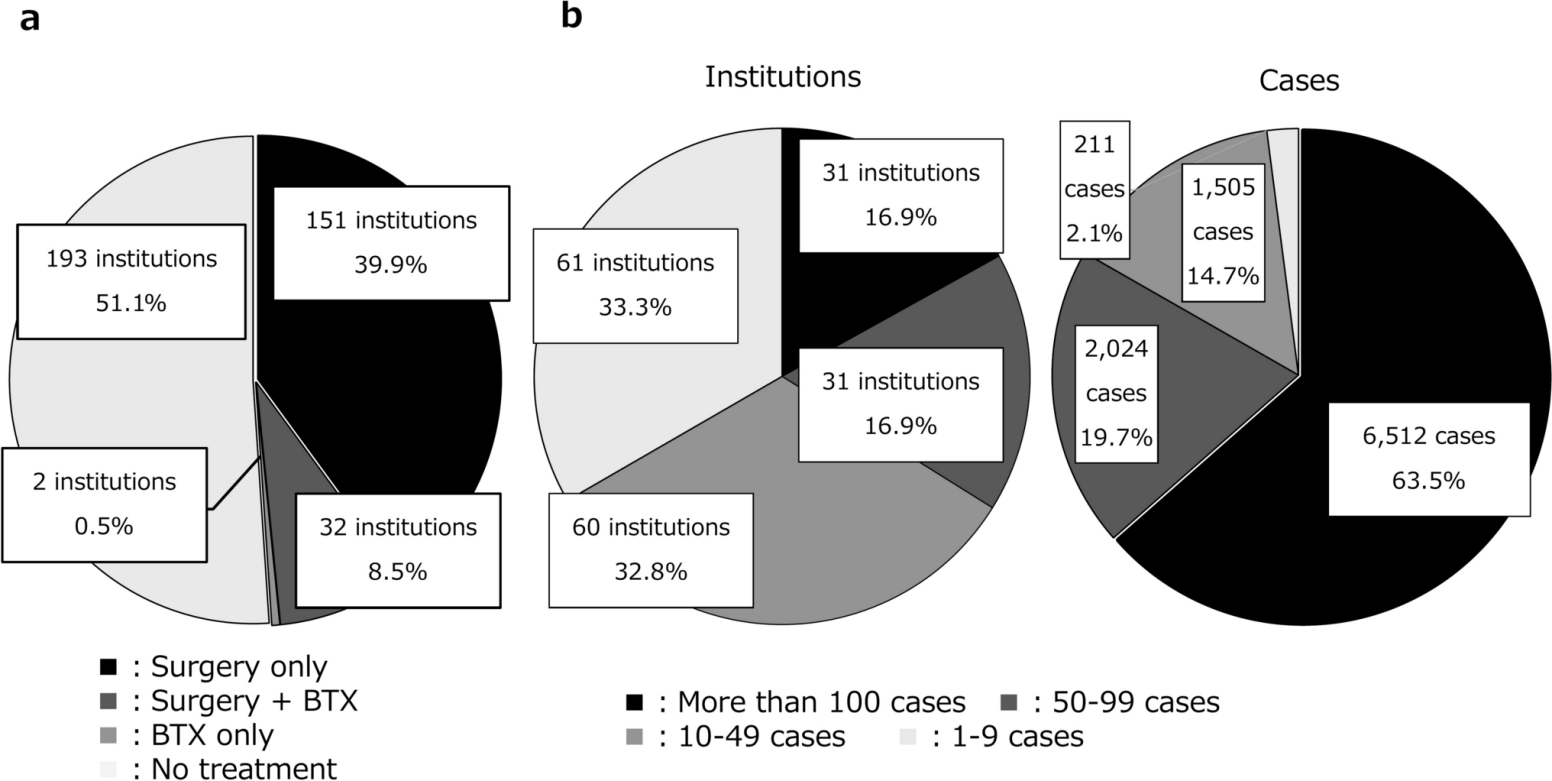
Institutions providing strabismus treatment (surgery and BTX therapy). **a** Proportions of the methods of strabismus treatment. Nearly half of the institutions in Japan provide strabismus treatment. **b** Proportions of the number of institutions and cases for strabismus surgeries. Approximately 34% of the institutions performed > 50 strabismus surgeries in a year, accounting for 83% of all cases. *BTX* botulinum toxin

Among the 1686 institutions that received the questionnaire, 376 institutions (22%) participated in the survey, including 64 institutions (79%) from all 81 university hospitals in Japan. Strabismus surgery and BTX therapy were performed at 185 institutions (49%). In total, 10,252 strabismus surgeries were performed at 183 institutions, and 515 BTX therapy sessions, at 34 institutions, yielding 10,767 cases in which strabismus treatment was given (Fig. 1a). Only surgery was performed in 151 (40%) of the institutions, surgery and BTX therapy in 32 (8%) of the institutions, and only BTX therapy in 2 (1%) of the institutions. The number of institutions and number of operated cases are shown in Fig. 1b.

Regarding the distribution of the institutions where strabismus surgery was performed, 48.2% of all surgeries were performed in 4 prefectures and strabismus surgery was not performed in any of the institutions in 3 prefectures (Online Resource 3-a). The average number of strabismus surgeries performed was 0.7 per 10,000 population; the ratio was widely distributed (0–3.5) (Online Resource 3-b, 4).

### Strabismus surgery

#### Distribution of patients and treatment methods

**Fig. 2:**
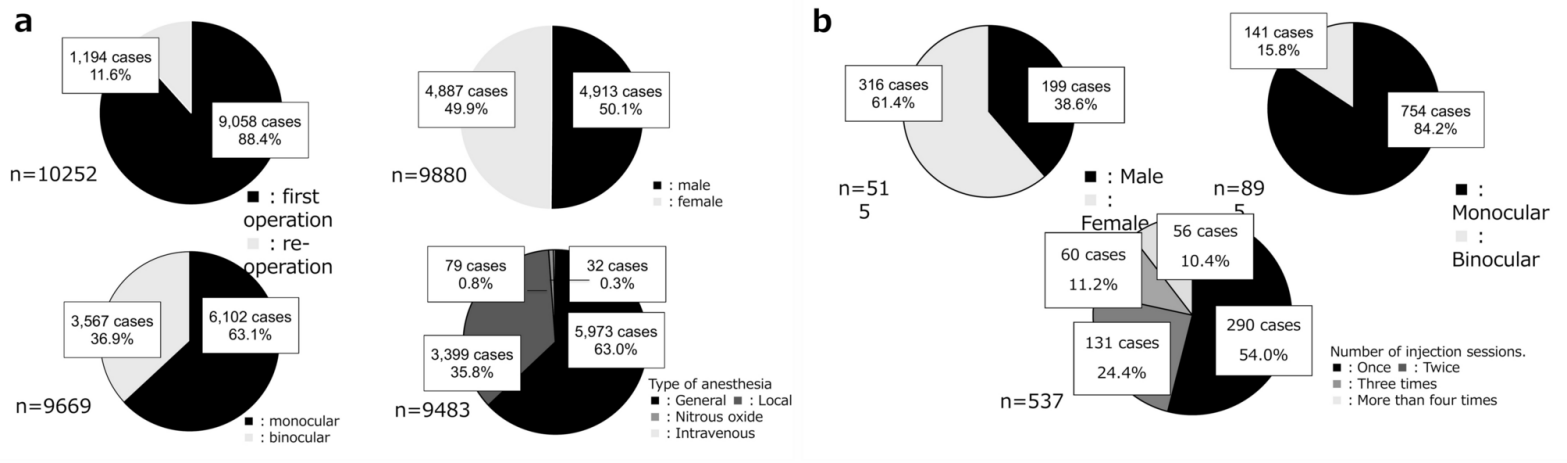
Distribution shown by the survey. **a** Surgery cases comprised 12% of reoperations. Among the total patients, 50% were men. A total of 63% of patients underwent unilateral surgeries, and 63% of patients underwent surgery under general anesthesia. **b** BTX therapy was administered to 61% of women. Nearly 84.2% BTX therapy cases received treatment in 1 eye. BTX therapy was administered once in 54% of cases and more than 4 times in 10% of cases. *BTX* botulinum toxin

Regarding sex, 4913 (50%) of the patients were male, and 4887 (50%), female. Strabismus surgery was performed for the first time in 88% of the patients, whereas 12% underwent reoperations. Surgery was performed on almost the same number of male and female patients. General anesthesia was frequently administered (63%), followed by local anesthesia including topical, sub-Tenon, and retrobulbar anesthesia without intravenous sedation (Fig. 2a).

#### Age at the time of strabismus surgery

**Fig. 3:**
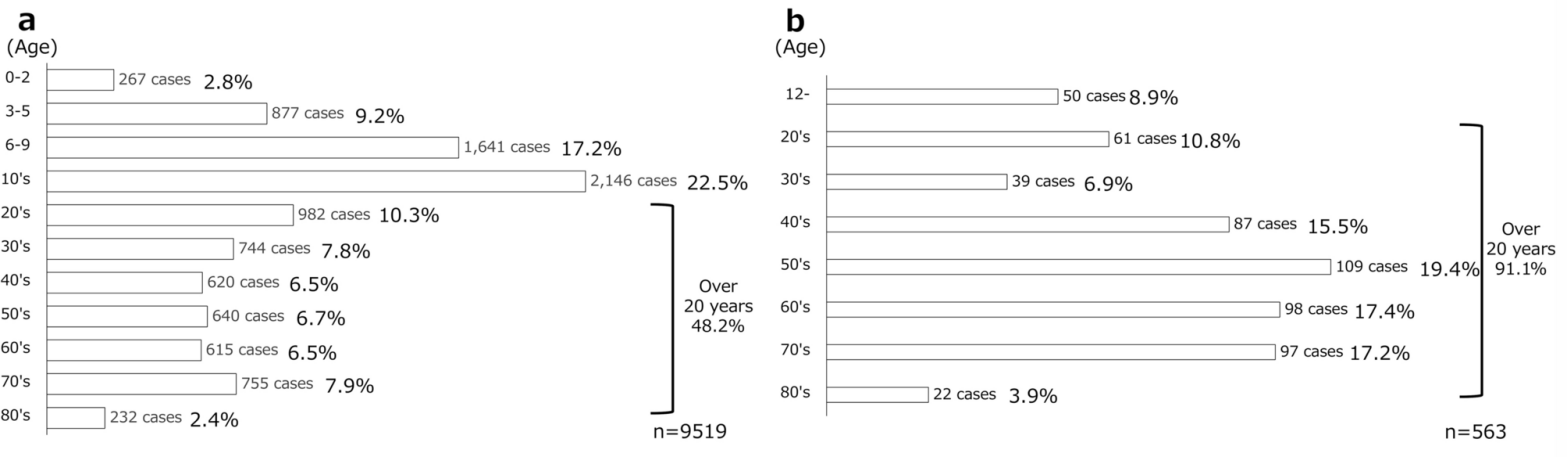
Proportions of patients by age at surgery. **a** Most of the patients were aged 10–19 years (“10s”) at the time of surgery (23%), and 52% were aged older than 20 years. In addition, 4588 of the patients (48%) were aged 20 years or older, and 1602 (17%), aged 60 years or older. *BTX* botulinum toxin. **b** The majority of patients receiving BTX treatment were in their 50s (20%), and 91% were aged ≥ 20 years

Patients aged 10–19 years accounted for the highest proportion of cases (2146 of 9519 [22.5%]). The number of patients aged ≥ 20 years was 4588 (48.2%), whilst that of patients aged ≥ 60 years was 1602 (16.8%) (Fig. 3).

#### Strabismus types and treatment methods

**Fig. 4:**
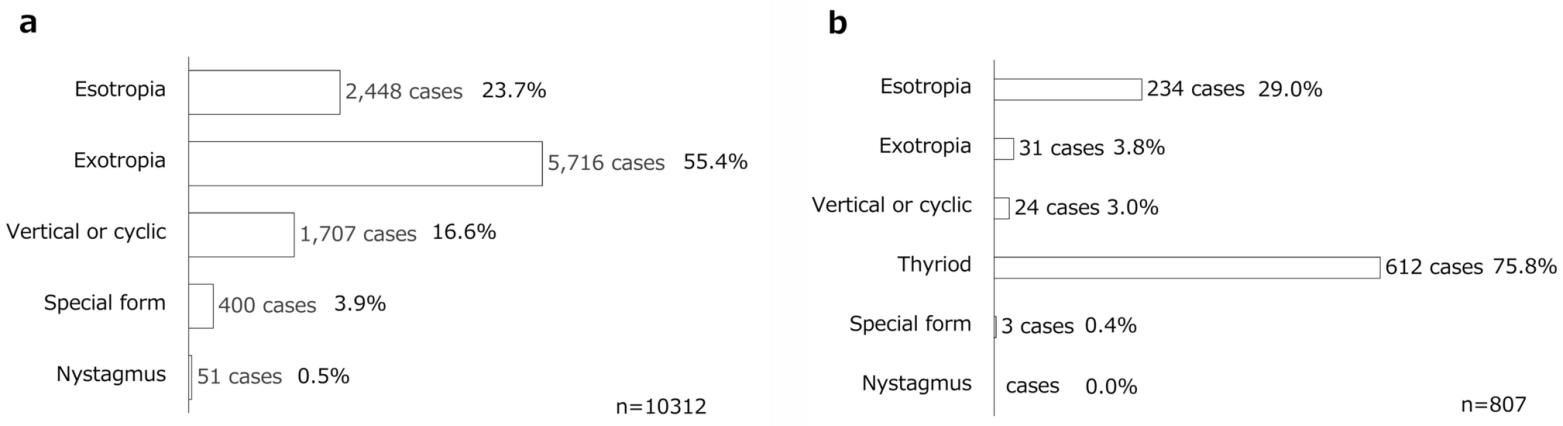
Proportions of the types of strabismus. **a** Associated surgery. Exotropia accounted for the majority of surgical cases (55%) and occurred twice as frequently as esotropia. **b** Associated BTX therapy. Thyroid eye disease accounted for the majority of cases receiving BTX treatments, at 75.8%. *BTX* botulinum toxin

**Fig. 5.**
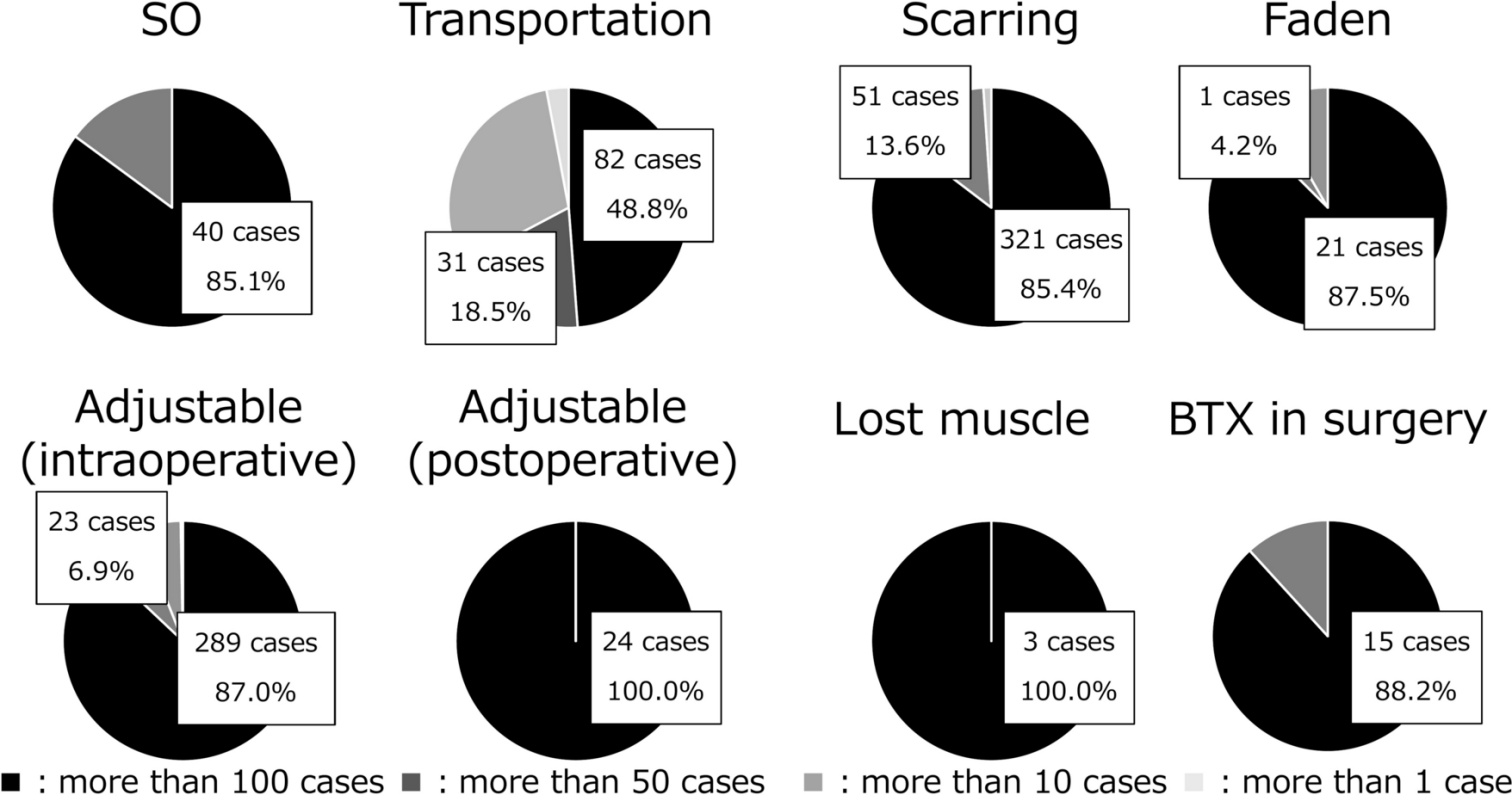
Proportions of complex cases among surgical procedures in institutions performing > 100 cases a year. In institutions performing more than 100 surgeries per year, 80% of all complex surgeries were performed. *SO* surgery for superior oblique muscle, *Adjustable* adjustable suture technique, *BTX* botulinum toxin

Exotropia (XT) was the most common type of strabismus (55%), followed by esotropia (ET) (24%). The number of surgeries for patients with XT was twice that for patients with ET (Fig. 4). In institutions where more than 100 surgeries were performed per year, 80% of all complex surgeries were performed for the superior oblique muscle (85%), scarred muscle (85%), and lost muscle (100%) (Fig. 5).

#### BTX therapy

Of the 34 centers where BTX therapy was performed, 268 cases (52%) were performed at the institution that had the highest number of BTX cases, 97% of which were thyroid eye disease (TED); 98% of the patients treated for strabismus at this institution had thyroid eye disease. In the two institutions where strabismus surgery was not performed, the number of cases was 93 (18%).

BTX was administered more frequently in females (61%) than in males (39%). Regarding laterality, 84% of the patients received BTX in 1 eye, whereas 16% received BTX in both eyes on the same day. The frequency of administration is shown in Fig. 2b; the age distribution of the patients is shown in Fig. 3. TED was the disease most frequently (76%) treated with BTX, followed by ET (29%) and XT (4%) (Fig. 4). Regarding the muscles treated with BTX, it was administered to 1 muscle in 60% of the patients, more than 2 muscles in 2% of the patients, and to scarred muscles owing to TED in 39% of the patients.

## Discussion

The survey highlighted several key trends in the current treatment of strabismus in Japan.

### Localization of strabismus treatment

The regional concentration of surgeries, with 48% performed in only 4 prefectures and none in 3 prefectures, suggests significant geographical disparities in access to strabismus treatment. Tokyo, with its high number of surgeries per capita, highlights the concentration of specialized services in urban areas. In addition, the result showing that 80% of complex strabismus surgeries were performed in institutions where more than 100 surgeries were performed suggests that strabismus treatment is performed only in specialized institutions and unevenly distributed.

### Trends and changes from 2013 to 2021

Considering trends in strabismus treatment in Japan, we compared the results of this survey with those of the first national survey, conducted in 2013. In 2013, in 57 institutions (18%) more than 50 surgeries were performed annually, whereas in 2021, this number had increased to 61 institutions (34%). This change suggests an increase in high-volume surgical centers. In addition, the number of strabismus surgeries decreased from 10,671 (2013) to 10,252 (2021). However, when combined with BTX therapy, the number of strabismus treatments for both was almost the same—10,671 (2013) and 10,767 (2021) cases—suggesting the possibility of localization of institutions set for strabismus treatment.

The most common age group for strabismus surgery in both surveys was 10–19 years (2021, 23%; 2013, 20%). However, the number of patients aged ≥ 20 years significantly increased (2021, 48%; 2013, 41%). Particularly, the number of patients aged 20–29 years increased 1.6-fold. XT was the most frequently treated condition, but its proportion decreased from 58% (2013) to 55% (2021), whereas the proportion of ET increased from 22% (2013) to 24% (2021).

The proportions of complex surgeries performed, compared with the overall cases, increased in institutions performing > 100 cases a year, from 56% (2013) to 85% (2021) for the superior oblique muscles, from 60% (2013) to 85% (2021) for scarred muscles, and from 70% (2013) to 100% (2021) for lost muscles. In 2021, 80% of complex surgeries were performed at institutions with more than 100 cases, indicating that strabismus surgery is becoming more specialized by institution.

### Factors influencing changes in age and strabismus disease trends

First, in regard to the increase in strabismus surgeries by age, particularly among adults aged older than 20 years, based on population ratios, a characteristic unique to Japan is the demographic shift from 2013 to 2021. The percentage of older adults (aged ≥ 65 years) increased from 25.1% to 28.8%, whilst the percentage of children (aged 0–14 years) decreased from 13.1% to 11.8%, from 2013 to 2021, highlighting an aging population and declining birth rates. By comparison, the elderly population in 2021 in Germany was 22.1% (children, 13.6%); in the United States, 16.9% (children, 18.3%); and in China, 12.6% (children, 17.1%) [9, 10]. Thus, an aging population and a low birthrate are the major factors underlying the change in age distribution [11]. Meanwhile, the increase in the rate of strabismus surgery among older adults may be attributed to the increasing healthy life expectancy and increased awareness regarding the treatment of age-related changes in strabismus disorders, including sagging eye syndrome (SES) [12–16]. The number of patients undergoing strabismus surgery for ET increased from 22.1% to 22.7%. SES was reported in 2008 as strabismus due to an age-related change in orbital connective tissue, and its awareness has recently spread to Japan [17, 18]. The increase in older patients undergoing strabismus surgery could be due to an aging population and increased awareness of age-related strabismus conditions such as SES.

### TED was the most common BTX therapy, followed by ET

A single institution accounted for 52% of cases of BTX administration, mostly for TED, indicating the concentration of BTX therapy in a few institutions and that the spread of the technique is still underway. In a previous report, surgery was deemed unnecessary in 32% of patients with TED after BTX treatment, whilst surgery was performed in 27% of patients, resulting in decreased correction [19]. Patients with TED who received preoperative BTX treatment may report an improvement in subjective symptoms and postoperative outcomes [20, 21]. In addition, 18% of all patients who received BTX therapy were from 2 institutions that did not perform strabismus surgery. These 2 institutions referred patients who did not respond adequately to BTX therapy to institutions that performed strabismus surgery.

As noted in the section comparing strabismus treatment in 2013 and 2021, although the total number of surgeries decreased, the total number of patients receiving treatments (surgery and BTX therapy) remained the same as that in 2013. In addition, regarding the number of strabismus surgeries for special strabismus with TED in 2013, the percentage was also calculated in this study as special strabismus combined with TED. The results showed that the percentage of special strabismus decreased from 5.0% to 4% in 2013, indicating that BTX therapy, particularly for TED, may decrease the need for strabismus surgery. Regarding the number of cases of ET, which was second only to thyroid eye disease, infantile ET and partially adjustable ET are conditions that occur in patients aged younger than 12 years, the target age for administration. Additionally, considering that these drugs were administered to patients aged older than 12 years, it is expected that many of these treatments were given for ET due to use of digital devices, which has been reported recently, and abducens nerve palsy [22].

## Limitations

The survey has limitations, including a lower response rate in 2021 than that in 2013 and the possibility of response bias due to the fact that it was a questionnaire survey. Additionally, although the impact of the coronavirus disease 2019 pandemic on strabismus surgery rates was not directly assessed, it likely influenced pediatric and elective surgeries.

This nationwide questionnaire survey revealed changes in the patient population and the treatment of strabismus in 2021 in Japan. It also showed that the concentration of strabismus treatment was a problem; dealing with regional disparities is an important objective.

## Supplementary Information

Below is the link to the electronic supplementary material.

Supplementary file1 (DOCX 36 KB)

Supplementary file2 (DOCX 36 KB)

Supplementary file3 (PPTX 1076 KB)

Supplementary file4 (DOCX 35 KB)

## Data Availability

The raw data were generated at the International University of Health and Welfare, Atami Hospital. All the data generated or analyzed during this study are included in this published article and its supplementary information files. The derived data supporting the findings of this study are available from the corresponding author on request.
