# Supplementary material for: 2021 trends in the treatment of patients with strabismus in Japan
Source: Jpn J Ophthalmol. 2024 Dec 16;69(1):10–6. doi: 10.1007/s10384-024-01144-5 (PMC11821698; doi:10.1007/s10384-024-01144-5)
Supplement: Supplementary file 1 — Supplementary file1 (DOCX 36 KB) [file 10384_2024_1144_MOESM1_ESM.docx]

Online Resource 1. Survey questions about surgeries.

| 1. No. of surgeries | Initial/repeated |  | 6. Type of strabismus | Esotropia |
| --- | --- | --- | --- | --- |
| 2. Treated eye | Monocular/binocular |  |  | Exotropia |
| 3. Anesthesia | General anesthesia |  |  | Thyroid eye disease |
|  | Local anesthesia |  |  | *Special forms |
|  | Intravenous |  |  | Nystagmus |
|  | Nitrous oxide |  | 7. Surgical procedures | One rectus muscle recession or resection |
| 4. Sex | Male/female |  |  | Two or more rectus muscle recessions or resections |
| 5. Age at surgery | 0–2, 3–5, 6–9 |  |  | IO muscle surgery |
|  | 10s |  |  | SO muscle surgery |
|  | 20s |  |  | Transposition procedure |
|  | 30s |  |  | Previous ocular or scarring |
|  | 40s |  |  | Faden operation |
|  | 50s |  |  | Adjustable suture (intraoperative) |
|  | 60s |  |  | Adjustable suture (postoperative) |
|  | 70s |  |  |  |
|  | 80s |  |  |  |

IO, inferior oblique muscle; SO, superior oblique muscle; BTX, botulinus toxin

* Special forms include Duane syndrome, Brown syndrome, general fibrosis, strabismus fixus, orbital floor fracture, and systemic associations such as myasthenia gravis.
