# Supplementary material for: 2021 trends in the treatment of patients with strabismus in Japan
Source: Jpn J Ophthalmol. 2024 Dec 16;69(1):10–6. doi: 10.1007/s10384-024-01144-5 (PMC11821698; doi:10.1007/s10384-024-01144-5)
Supplement: Supplementary file 2 — Supplementary file2 (DOCX 36 KB) [file 10384_2024_1144_MOESM2_ESM.docx]

Online Resource 2. Survey questions about BTX therapies.

| 1. Frequency | Once |  | 5. Type of strabismus | Esotropia |
| --- | --- | --- | --- | --- |
|  | Twice |  |  | Exotropia |
|  | Thrice |  |  | Vertical or cycloptic |
|  | More than 4 times |  |  | Thyroid eye disease |
| 2. Treated eye | Monocular/binocular |  |  | *Special forms |
| 3. Sex | Male/female |  |  | Nystagmus |
| 4. Age at injection | 12–19 |  | 6. Method of treatment | One rectus muscle |
|  | 20s |  |  | Two or more rectus muscle |
|  | 30s |  |  | IO |
|  | 40s |  |  | SO |
|  | 50s |  |  |  |
|  | 60s |  |  |  |
|  | 70s |  |  |  |
|  | 80s |  |  |  |

IO, inferior oblique muscle; SO, superior oblique muscle; BTX, botulinus toxin

* Special forms include Duane syndrome, Brown syndrome, general fibrosis, strabismus fixus, orbital floor fracture, and systemic associations such as myasthenia gravis.
