# Supplementary figures and images for: 2021 trends in the treatment of patients with strabismus in Japan
Source: Jpn J Ophthalmol. 2024 Dec 16;69(1):10–6. doi: 10.1007/s10384-024-01144-5 (PMC11821698; doi:10.1007/s10384-024-01144-5)

## Slide 1
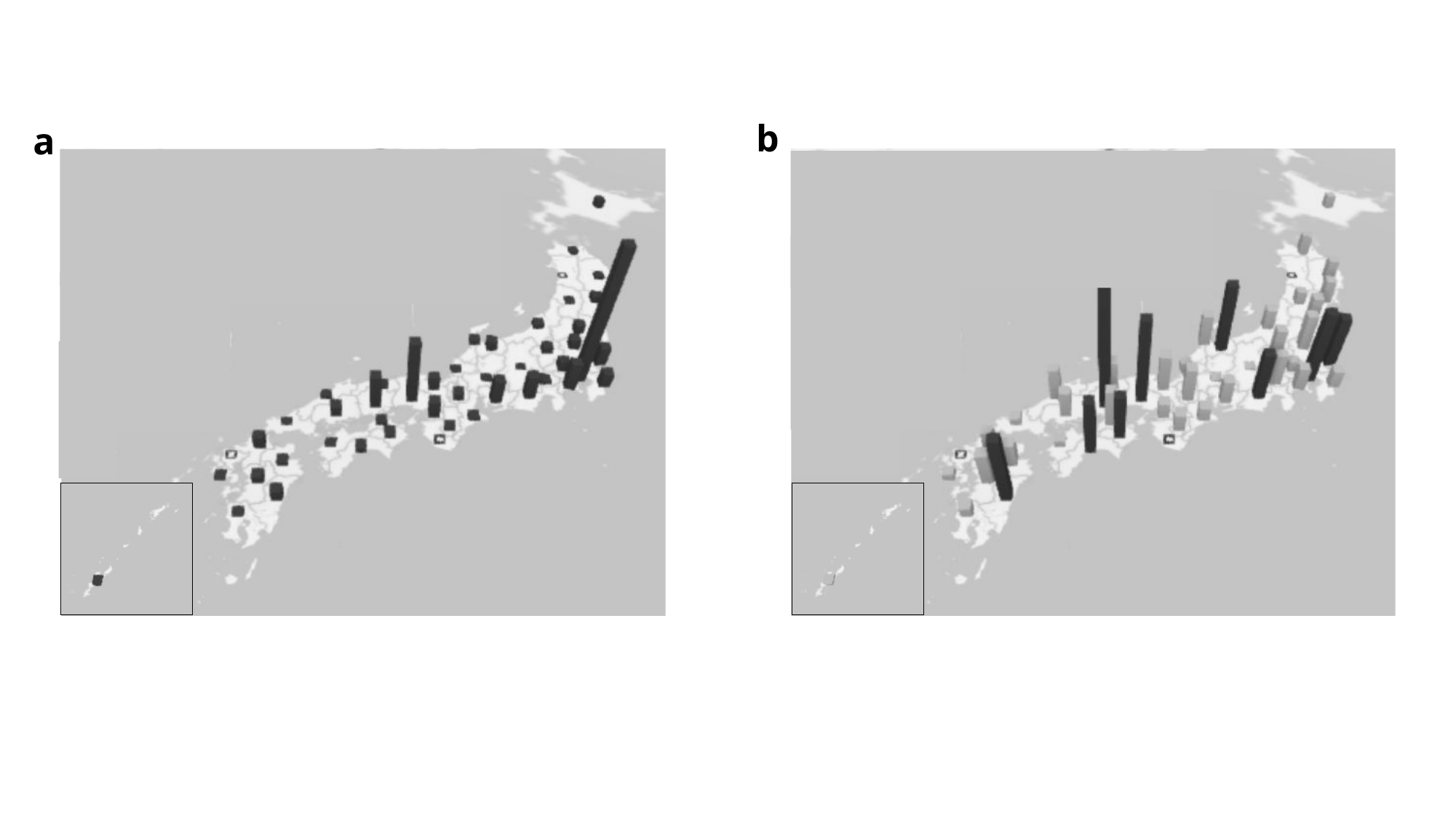

b
a

Supplement: Supplementary file 3 — Supplementary file3 (PPTX 1076 KB) [file 10384_2024_1144_MOESM3_ESM.pptx]
