# Supplementary material for: 2021 trends in the treatment of patients with strabismus in Japan
Source: Jpn J Ophthalmol. 2024 Dec 16;69(1):10–6. doi: 10.1007/s10384-024-01144-5 (PMC11821698; doi:10.1007/s10384-024-01144-5)
Supplement: Supplementary file 4 — Supplementary file4 (DOCX 35 KB) [file 10384_2024_1144_MOESM4_ESM.docx]

Online Resource 4. Top 10 of total number of surgeries and cases per 10,000 people.

| Rank | Prefectures | No. cases | Prefectures | No cases.  per 10,000 population |
| --- | --- | --- | --- | --- |
| 1 | Tokyo | 2578 | Okayama | 3.5 |
| 2 | Hyogo | 1203 | Hyogo | 2.2 |
| 3 | Okayama | 650 | Miyazaki | 1.8 |
| 4 | Kanagawa | 528 | Tokyo | 1.8 |
| 5 | Aichi | 457 | Toyama | 1.7 |
| 6 | Shizuoka | 445 | Kochi | 1.5 |
| 7 | Ibaraki | 371 | Ibaraki | 1.3 |
| 8 | Osaka | 356 | Shizuoka | 1.2 |
| 9 | Chiba | 280 | Tokushima | 1.2 |
| 10 | Kyoto | 238 | Kagawa | 1.0 |

*Ranked prefecture in both were painted slant stripe.
